# Neuroanatomical correlates of forgiving unintentional harms

**DOI:** 10.1038/srep45967

**Published:** 2017-04-06

**Authors:** Indrajeet Patil, Marta Calò, Federico Fornasier, Liane Young, Giorgia Silani

**Affiliations:** 1 Scuola Internazionale Superiore di Studi Avanzati, Neuroscience Sector, Trieste, Italy; 2 Department of Psychology, Harvard University, Cambridge, MA, USA; 3 University of Trieste, Trieste, Italy; 4 Department of Psychology, Boston College, Boston, USA; 5 Department of Applied Psychology: Health, Development, Enhancement and Intervention, University of Vienna, Austria

## Abstract

Mature moral judgments rely on the consideration of a perpetrator’s mental state as well as harmfulness of the outcomes produced. Prior work has focused primarily on the functional correlates of how intent information is neurally represented for moral judgments, but few studies have investigated whether individual differences in neuroanatomy can also explain variation in moral judgments. In the current study, we conducted voxel-based morphometry analyses to address this question. We found that local grey matter volume in the left anterior superior temporal sulcus, a region in the functionally defined theory of mind or mentalizing network, was associated with the degree to which participants relied on information about innocent intentions to forgive accidental harms. Our findings provide further support for the key role of mentalizing in the forgiveness of accidental harms and contribute preliminary evidence for the neuroanatomical basis of individual differences in moral judgments.

When humans make moral judgments, one critical input is information about intent. Did she spill the hot coffee on her colleague on purpose? Did he step on his competitor’s foot by accident? Similarly, the common law tradition relies on presence of culpable mental states (*mens rea*) for criminal conviction. Much recent work in moral psychology and cognitive neuroscience has explored the psychological and neurofunctional basis of intent processing for moral judgment. In the current study, we extend this literature by exploring the neuroanatomical correlates of this process. Behavioral work shows that when intent and outcome information conflict, people primarily rely on information about intent, an effect observed in older children and adults across cultures^1^,^2^,^3^. Representations of others’ mental states are constructed by *Theory of Mind* (ToM) or mentalizing, the process of inferring representational content (e.g., beliefs, desires, knowledge, intentions) from observing others’ in order to explain and predict their behavior^4^,^5^. This capacity is neurally implemented in a specific network consisting primarily of the bilateral temporoparietal junction (TPJ), ventromedial prefrontal cortex (vmPFC), dorsomedial prefrontal cortex (dmPFC), temporal poles (TP), superior temporal sulcus (STS), and precuneus/posterior cingulate cortex (PC/PCC)^4^.

There is ample amount of evidence that shows an overlap between the moral reasoning network and ToM network, as highlighted by a recent meta-analysis^6^. Latest work has also begun attributing specific functions that various regions in ToM network may play during moral evaluations. For example, the encoding of mental states occurs very early during information processing in the right TPJ^7^, left TPJ^8^, and amygdala^9^. The rTPJ, dmPFC, and PCC are also involved in integrating belief states^10^,^11^ with other morally relevant pieces of information (e.g., consequences) to construct final moral judgments^12^,^13^,^14^,^15^. On the other hand, the dmPFC is involved in encoding the valence (harmful or neutral) of the beliefs^10^. Individual differences in both the overall magnitude of activity and the spatial pattern of activity in rTPJ have been repeatedly associated with the degree to which information about innocent intentions is used to forgive agents who cause accidental harms^16^,^17^,^18^. Additionally, disrupting activity in rTPJ via transcranial magnetic stimulation (TMS) leads to a more lenient assessment of attempted harms^19^, while enhancing this activity via transcranial direct-current stimulation (tDCS) leads to reduced blame for accidents^20^.

In summary, this work shows that neural activity in the ToM network, in general, is involved in encoding and integrating the information about mental states of actors involved in moral situations. This network thereby also underpins cognitive basis of how people forgive^21^,^22^ accidents based on innocent intentions and condemn attempted harms based on malicious intentions.

Although extant work delineates the neurofunctional correlates of intent-based moral judgements, the neurostructural basis of this process remains sparsely studied. Previous morphometry studies relating to moral cognition have examined how regional variation in brain structure relates to individual differences in endorsed moral values^23^, moral development^24^, group-focused moral foundations^25^, injustice sensitivity^26^, indirect reciprocity^27^, prosocial behavior^28^,^29^,^30^,^31^, and moral judgments in clinical populations^32^,^33^. To our knowledge, only one prior voxel-based morphometry (VBM) study has investigated this issue in a small sample (*n* = 19), recruited as controls for comparison with a neurological population^32^. This study found correlation patterns for accidental harm in regions typically associated with ToM, namely PC, vmPFC, and dorsolateral prefrontal cortex (dlPFC), although no claim about their functional properties was made in this work.

Given the consistent prior evidence from fMRI and VBM studies implicating the ToM network in forgiving accidental harms^16^,^17^,^18^, we focused our attention on this context: we predicted that volumetric differences in regions belonging to the ToM network will explain variation in moral judgments of accidental harms.

## Methods and Materials

### Participants

A total of 50 healthy community members (32 female; 41 right-handed) were recruited to participate and financially compensated for their time and travel. Average age was 23.06 years (SD = 3.08), with a range of 18 to 35. All participants gave written informed consent. This study was conducted according to the principles in the Declaration of Helsinki, approved by the Ethics Committee of the Hospital ‘Santa Maria della Misericordia’ (Udine, Italy), and was carried out in accordance with the approved guidelines. All participants provided written informed consent before any study procedure was initiated.

Rule-out criteria for participation included Italian as a secondary language, presence of a diagnosed psychiatric illness and/or history of psychiatric treatment, history of significant neurological illness or brain injury, and current usage of psychoactive drugs. All participants were screened for neurological condition and MRI contraindications in two-step checks, during a pre-scanning telephone interview and before entering the scanner. All data (structural, functional, and behavioral) from one participant were excluded from the final analysis as this participant was consuming clinically prescribed psychoactive drugs and divulged this information only during the post-scan debriefing. Thus, structural and behavioral data were available for 49 participants. Functional data for the moral judgment task from two participants were removed due to excessive head motion, four participants due to high collinearity in regressors, and data from one additional participant could not be collected due to technical error (final *n* = 42).

### Experimental stimuli and procedure

Participants performed a moral judgment task in the scanner. Additionally, they completed a ToM localizer task. The order of tasks (described in detail below) was counterbalanced across participants. Although the functional data from the moral judgment task are described in detail in a different manuscript, we offer a brief summary of data acquisition and analysis procedures, given our inclusion of exploratory functional data analysis based on our VBM analysis results (see below).

#### Moral judgment task

Experimental stimuli were text-based scenarios. Scenarios were largely adapted from previous studies^1^,^19^ and translated into Italian (see Supplementary Text S1 for more details). There were four variations of 36 unique scenarios for a total of 144 stories. The four variations were the result of a 2 × 2 within-subjects design where the factors *belief* (neutral, negative) and *outcome* (neutral, negative) were independently varied. Each participant saw one variation of each scenario, for a total of 36 stories.

Each scenario lasted for 32 s and consisted of four cumulative segments (each lasting for 8 s): (*i*) *background:* this segment was common to all variations and provided context for the action; (*ii*) *foreshadow:* this segment foreshadowed the outcome as neutral or harmful; (*iii*) *mental state information:* this segment provided information about the agent’s neutral or harmful belief; (*iv*) *consequence:* this final segment described the agent’s action and the outcome. We use the term *mental state information* instead of *belief* and *consequence* instead of *outcome* to avoid confusion as the latter terms represent factors of the experimental design, while the former represent story segments containing information about the agent’s beliefs and the nature of the outcome, respectively. We provide an example of one story below (called “Rabid dog”, presented here in the accidental harm condition):

#### Background

Chiara works at the pound. Several new dogs have just come in. A lady comes in, interested in taking one of the new dogs home with her.

#### Foreshadow

The dogs are very sick with rabies and will make their owners sick too by biting them.

#### Mental state information

Chiara talks with one of the other people at the pound. Chiara thinks that the new dogs have been through a thorough health inspection and will make good pets.

#### Consequence

Chiara gives the lady one of the new dogs. It is infected with rabies and bites the lady on the neck on the very first day.

After reading each story, participants provided two types of moral judgments following prior work^1^, presented in randomized order across trials for each participant:

[1] *acceptability* - “How morally acceptable was [the agent]’s behavior?” (1: *Completely acceptable* to 7: *Not at all acceptable*);

[2] *blame* - “How much blame does [the agent] deserve?” (1: *None at all* to 7: *Very much*).

Each question was presented on the screen for 6 s, and participants could provide judgments using a 7-point Likert scale, on which the cursor could be moved using two fingers. Since these two judgments were highly correlated (neutral: *r* = 0.875, accidental: *r* = 0.863, attempted: *r* = 0.889, intentional: *r* = 0.617), they were averaged to form a single moral judgment per condition, indexing severity of moral condemnation.

#### ToM localizer task

**Figure 1:**
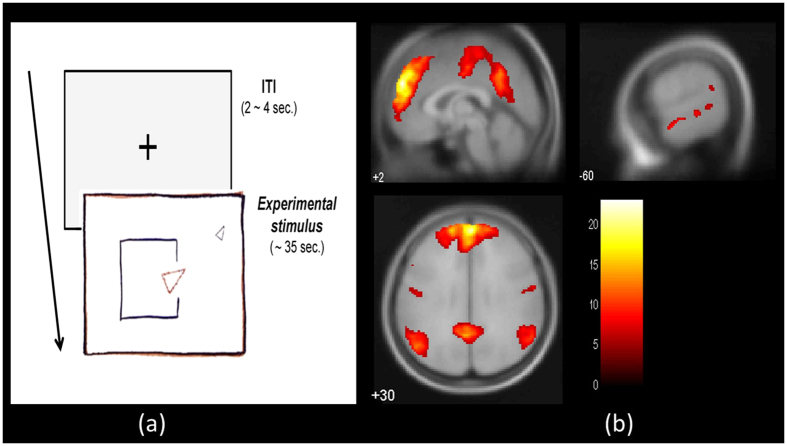
Schematics and results for the Theory of Mind (ToM) localizer task. (**a**) Participants watched animations involving two triangles interacting with each other in either a meaningful way (experimental) or in a random manner (control). (**b**) The ToM network localized at the group level using group independent component analysis (gICA) on the ToM functional localizer task. Regions including the bilateral temporoparietal junction (TPJ), sections of medial prefrontal cortex (mPFC), temporal poles (TP), superior temporal sulcus (STS), and precuneus (PC) formed a functionally connected network. Statistical maps represent *t-*values thresholded at a voxel-wise threshold of *p* < 0.05 (FWE-corrected, height threshold: *t* = 5.62) and extent threshold of 10 voxels. The color bar denotes *t*-values. A similar network was observed in GLM-based analysis of the data (see Supplementary Text S2). Abbreviation - ITI: intertrial interval.

In order to localize functional regions involved in mental state attribution, we used the animated shapes paradigm based on the Frith-Happé animations^34^, a reliable and sensitive method for eliciting spontaneous mentalizing^35^. The stimuli consisted of eight animations (four per condition) featuring two triangles (a big red triangle and a small blue triangle) moving on a white background. Stimuli were matched for overall shape, speed, and orientation changes as closely as possible. ToM animations featured complex scripts in which triangles interacted in a way that gave semblance of bluffing, mocking, persuading, and surprising. By contrast, control animations comprised of random, billiard ball-like motion of the two triangles. The length of videos was matched for the two types of animations (on average 34–35 s), and the order of condition appearance was randomized. While there was no active task, we ensured that participants closely attended to the videos by telling them there would be questions at the end of the task (in reality, there were no questions asked). Intertrial interval (ITI) was randomly jittered with an average of 2 s (jitter range: 0–2 s) (Fig. 1(a)).

### Functional scan acquisition

All fMRI scans were acquired using a 3 T Philips Achieva scanner equipped with an 8-channel head coil. High-resolution structural images were acquired as 180 T1-weighted transverse images (0.75 mm slice thickness). Functional images were acquired in interleaved manner using a T2*-weighted echoplanar imaging (EPI) sequence with 33 transverse slices covering the whole brain with the following parameters: slice thickness = 3.2 mm; interslice gap = 0.3 mm; repetition time (TR) = 2000 ms, echo time (TE) = 35 ms; flip angle = 90°, field of view = 230 × 230 mm^2^; matrix size = 128 × 128, SENSE factor 2. The slices were oriented at a 30° oblique angle to the AC-PC.

### fMRI data analysis

Data were preprocessed with SPM12 (Wellcome Department of Imaging Neuroscience, London, UK) running on MATLAB R 2013 a. Each subject’s data were motion-corrected and then normalized onto a common stereotactic space (the MNI template). Data were then smoothed by using a Gaussian filter (FWHM = 6 mm at first-level) and high-pass-filtered.

### ToM localizer task

For technical reasons (see Supplementary Text S2), we preferred model-free group independent component analysis (gICA) over (GLM) to analyze the ToM localizer task data (although same results were obtained with GLM analysis, see below). ICA is a model-free analysis technique that allows one to characterize the spatio-temporal structure of the data. ICA identifies mutually independent sets of regions (component) exhibiting high within-component functional connectivity^36^.

We performed spatial gICA on preprocessed functional datasets (*n* = 49) of equal length using the GIFT toolbox (v4.0, http://mialab.mrn.org/software/gift/; Calhoun, Liu, & Adalı^37^) to localize the ToM network. The component corresponding to the ToM network was identified (for full details, see Supplementary Text S2) and the coordinates for the network were derived from a one-sample Student’s *t*-test. Statistical significance was assessed using an FWE value of *p* < 0.05 corrected for the whole-brain volume at the cluster-level (*k* = 10) The regions thus localized were saved as thresholded binary maps to be used as an inclusive ToM mask for the VBM analysis. We note that the key nodes of the ToM network (dmPFC, vmPFC, PC/PCC, bilateral TPJ, bilateral STS, TP) were observed in both model-free ICA and model-based GLM analyses (See Supplementary Text S4).

### Structural scan acquisition

High-resolution structural images were acquired as 190 T1-weighted transverse images with a 3D ultrafast gradient echo sequence on a 3T Philips Achieva scanner at the Hospital ‘Santa Maria della Misericordia’ (Udine, Italy) equipped with an 8-channel SENSE head coil. The following parameters were used: voxel size = 1 × 1 × 1 mm, slice thickness = 1 mm, TR/TE = 8.2/3.7 ms, matrix size = 240 × 240 mm, field of view = 19 cm, flip angle = 8°, no overcontiguous slices.

### Voxel-based morphometry

For reporting VBM analyses details, we have followed a set of prior guidelines^38^,^39^. Additional technical details are provided in Supplementary Text S3. Both preprocessing and statistical analysis of anatomical data were carried out using SPM12 according to procedures outlined in prior work^40^,^41^,^42^,^43^.

In order to account for the intensity inhomogeneities present in MR scans at high field strengths (≥3 T), bias field correction was applied during the segmentation procedure^44^. Each image was segmented into six different tissues classes (grey matter (GM), white matter (WM), cerebrospinal fluid (CSF), bone, other soft tissues, and air/background) using the modified unified segmentation approach^45^,^46^ implemented in SPM12. The Non-linear deformation field was estimated for each individual image such that tissue probability maps for each tissue class were best aligned. The segmented images were imported (only for GM and WM) both in native space and DARTEL space.

The segmented images (only GM and WM) were then iteratively registered via a fast diffeomorphic registration algorithm^47^ (DARTEL) to warp the GM and WM partitions into a study-specific template space representative of the average of all study subjects.

This process created a template image for the group of individuals and also estimated the nonlinear deformation flow fields that best aligned individual images together. This template image was then transformed to MNI stereotactic space (2 × 2 × 2 mm) using affine and non-linear spatial transformations to generate normalized, Jacobian-scaled (grey matter amount preserved, i.e.) GM images for each participant. These images were also simultaneously smoothed with an isotropic Gaussian kernel with FWHM of 10 mm. Note that these final smoothed images represent *absolute amount* of local GM at each voxel in the brain^48^,^49^. These smoothed normalized GM segments were then entered into a statistical model to conduct voxel-wise statistical tests and map significant effects.

The statistical analysis was carried out by fitting a GLM to the data. We included age, age^2^ (to model quadratic effects of age), handedness, and gender as nuisance covariates^50^,^51^. Since total intracranial volume (TIV) was entered as a global for proportional scaling, it was not included in the design matrix as a regressor. Repeating the same analysis by entering TIV values not as globals but as covariates produced similar results. Although no overall grand mean scaling was applied, we used global normalization by entering TIV values as globals (as recommended by Ridgway *et al*.^52^) in proportional scaling, which identifies specific regional changes that are not confounded by global differences.

Four separate regression models were created for each condition containing moral judgment score, age, age^2^, handedness, and gender as predictors and GMV as the dependent measure in each model. Two contrasts were created for each model that regressed local GMV on the moral judgment scores, one tracking positive association, while the other negative:

- Positive ([0, 1]; greater GMV associated with *increased* moral condemnation) and
- Negative ([0, −1]; greater GMV associated with *reduced* moral condemnation).

Importantly, the second-level analyses were restricted only to voxels contained in the inclusive mask derived from the ToM localizer task (see Fig. 1). Note that selection of voxels included in the mask was independent from the data used in the VBM analysis circumventing any circularity^53^. Given recent criticism of parametric cluster-level inference^54^,^55^, significant clusters were formed by employing the threshold-free cluster enhancement (TFCE) method (as implemented in TFCE toolbox (r95): http://dbm.neuro.uni-jena.de/tfce/). The TFCE is a cluster-based thresholding method that circumvents the problem of choosing an arbitrary cluster forming threshold (e.g., *p* < 0.001 (uncorrected) and *k* = 10) by taking a raw statistics image and producing an output image in which the voxel-wise values represent the amount of cluster-like local spatial support^56^. This also makes the TFCE inference fairly robust to non-stationarity in the data under varying smoothness levels, degrees of freedom and signal to noise ratios^57^,^58^. The TFCE image is then turned into voxel-wise *p*-values via a permutation-based non-parametric testing (5000 permutations were used). All group comparisons are reported at *p* < 0.05 after Family-wise Error (FWE) correction and, as recommended^59^,^60^, we report effect sizes in addition to statistic values.

## Results

### Behavioral data

**Figure 2:**
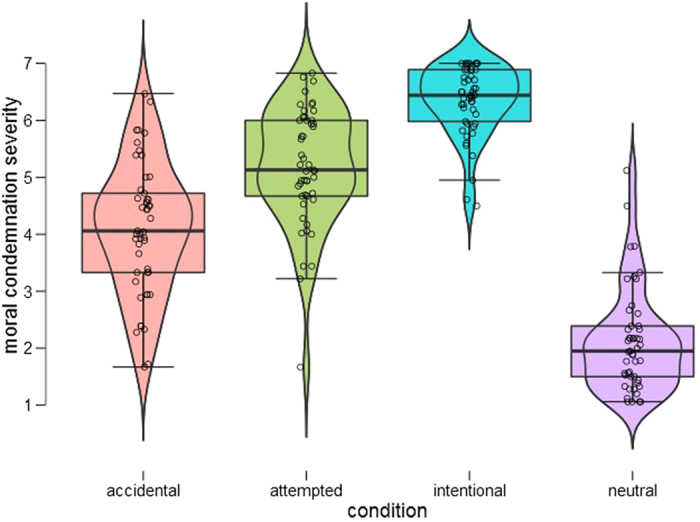
Moral condemnation ratings across conditions. Full distribution of moral condemnation ratings across conditions is shown using combination of box and violin plots^96^. Box plot within the violin plot contains thick black line for the median and the box indicates the interquartile range, while the added rotated kernel density plot shows the probability density of the data at different values. As can be seen, there was more variation in accidental and attempted harm cases, where intent and outcome was misaligned, as compared to neutral and intentional cases.

A 2-by-2 repeated measures ANOVA on moral judgment data revealed the expected main effects of intent (*F*(1, 48) = 217.778, *p* < 0.001, *pη*^2^ = 0.819), outcome (*F*(1, 48) = 122.012, *p* < 0.001, *pη*^2^ = 0.718), and their interaction (*F*(1, 48) = 30.393, *p* < 0.001, *pη*^2^ = 0.388). In other words, agents who acted with harmful intent or who produced a harmful outcome were condemned more severely than those acting with innocent intention or who produced a neutral outcome, respectively (Fig. 2; for descriptive statistics, see Supplementary Text S5). Additionally, the intent and outcome information interacted such that the degree to which the presence or absence of harmful consequence affected severity of moral condemnation depended on whether the intent was neutral or negative (greater difference in severity of moral judgment in accidental versus neutral comparison than intentional versus attempted comparison).

### Functional localizer results

The gICA on the ToM localizer task revealed a component consisting of the regions involved in mentalizing (see Fig. 1(b)), including bilateral TPJ, PC/PCC, dmPFC, TP, posterior STS, anterior STS, etc. (for more details on the component characteristic, see Supplementary Text S2) All VBM analyses were restricted to the binary mask comprising of voxels belonging to this network.

### Anatomical data

#### Neutral, attempted, and intentional harm conditions

No supra-threshold voxels were found for positive (greater GMV associated with *increased* moral condemnation) or negative (greater GMV associated with *reduced* moral condemnation) contrasts.

#### Accidental harm condition

**Figure 3:**
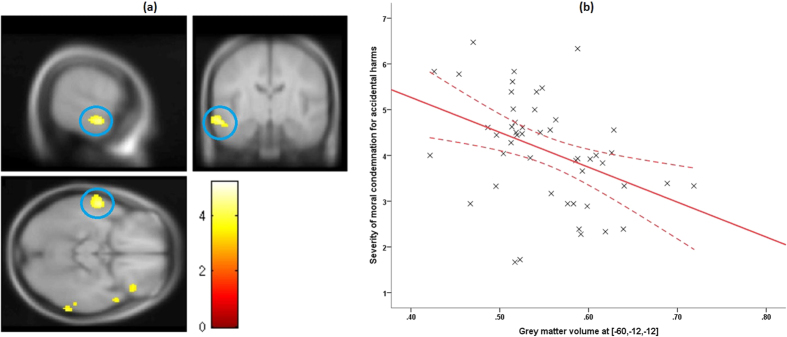
VBM results. (**a**) VBM result for accidental harm condition at group random effects analysis. Maps are thresholded at *p*(uncorrected) <0.001, *k* > 10, for illustrative purposes. The left anterior superior temporal sulcus (l-aSTS) is highlighted in the blue circle. The color bar denotes *t*-values. (**b**) A scatter plot illustrating the negative linear association between the grey matter volume (GMV) in l-aSTS (*ρ*(47) = −0.547, 95% CI [−0.726, −0.296], *p* < 0.001, *n* = 49, two-tailed) and the severity of moral condemnation of accidental harm, accounting for nuisance variables. The solid lines indicate a linear fit to the data, while the curved lines represent mean 95% confidence intervals for these lines. Extracted grey matter volume data presented in figures are non-independent of the statistical test used to find effect at this region and thus should *not* be used for effect-size estimates^97^. They are included here only as a visual aid for interpretation of results. Abbreviation - VBM: voxel-based morphometry.

Regression analyses revealed that more severe moral condemnation for accidental harm was associated with reduced GMV in left (*x* = −62, *y* = −12, *z* = −12; *β* = −0.0252, TFCE = 13.26; *k* = 202; *p*(FWE-corrected) = 0.002) anterior STS (aSTS) (see Fig. 3). Note that although we refer to this region here as aSTS^61^,^62^,^63^, other studies have also referred to the same region with the anatomical label “middle temporal gyrus (MTG)”^6^,^35^,^64^,^65^,^66^,^67^.

No supra-threshold voxels were found for positive contrast. Additionally, no suprathreshold voxels outside the localized ToM network were found for any contrast or for any condition in the whole-brain analysis.

### Data availability statement

Unthresholded VBM statistical maps of reported contrasts are available on Neurovault^68^ at the following address: http://neurovault.org/collections/1689/.

All the behavioral data are available at: https://osf.io/akx6a/.

### Post hoc exploratory functional data analysis

Given that functional data were also available for each participant, we decided to explore *post hoc* whether the extent to which innocent intentions are taken to mitigate condemnation for accidental harms is correlated with the functional activity at this region. Note that this region has not received much attention in prior work on this topic and thus we wanted to ascertain that functional activity in this region was predictive of moral judgments.

For ROI analysis, the data from spherical ROIs with a radius of 8 mm was extracted from l-aSTS at coordinates observed in the VBM analysis and was analyzed using the MarsBar toolbox for SPM (v0.44, http://marsbar.sourceforge.net/)^69^. Within the ROI, we extracted parameter estimates (*β*s) from all segments of interest (*mental state information, consequence, acceptability, blame*) for the accidental harm condition and correlated these with behavioral ratings, i.e., moral condemnation. Data defining ROIs were independent from data used in the repeated measures statistics^53^,^70^, and restricting analysis to a few ROIs reduced Type-I error by drastically limiting the number of statistical tests performed^71^.

**Figure 4:**
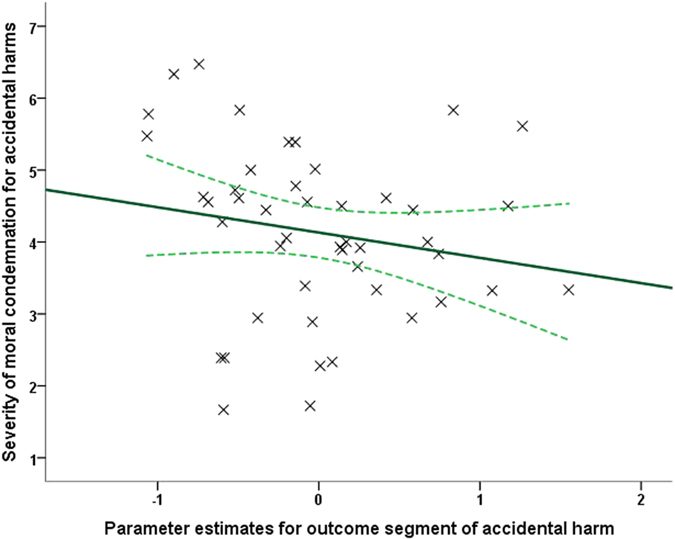
Brain-behavior correlation in fMRI data. A scatter plot illustrating the negative linear association (*ρ*(40) = −0.294, *p* = 0.029, *n* = 42, one-tailed) between the parameter estimates (*β*s) extracted from l-aSTS [−60, −12, −12] during the *consequence*/*outcome* segment (when outcome information was revealed) of the moral judgment task and the severity of moral condemnation of accidental harms. The solid lines indicate a linear fit to the data, while the curved lines represent mean 95% confidence intervals for this line.

Results revealed a negative correlation between parameter estimates extracted from the *consequence*/*outcome* segment and condemnation for accidents for l-aSTS (*ρ*(40) = −0.294, *p* = 0.029, *n* = 42, one-tailed) (Fig. 4). Note that we have used Spearman’s *rho* as our correlation measure, as it is more robust to univariate outliers^72^ and one-tailed correlation tests given our strong directional hypotheses^73^. None of the other correlations were significant (*p*s > 0.05, one-tailed).

## Discussion

In the present study, VBM was used to investigate whether inter-individual variation in intent-based moral judgments could be predicted from variation in the local GMV from regions belonging to the ToM network. We found that only variation in GMV in the l-aSTS, which was localized using an independent functional localizer task, could explain variance in moral condemnation of accidental harms: higher GMV was associated with increased tendency to forgive accidents (i.e., unintended harm to others). A similar profile was also observed in an exploratory analysis of functional data. These results raise several questions: What is the function of aSTS in the ToM network and its involvement in moral reasoning? What does the observed VBM effect signify? We discuss these issues below.

### The functional role of the l-aSTS

Over the last two decades, over 400 studies have investigated the neural basis of the ToM network, and this work has revealed a remarkably consistent set of brain regions, including bilateral TPJ, PC, sections of mPFC, bilateral STS, and TP. In the current study, we were able to delineate a network anatomically consistent with the meta-analytic definitions of the ToM network using a well-validated functional localizer task, featuring social animations, and VBM effect was found at left aSTS, which was part of the ToM network. Here, we speculate what psychological process this region may support during moral evaluations.

Quantitative meta-analyses and large-scale studies focusing on ToM tasks have consistently found aSTS/MTG^6^,^64^,^65^,^74^,^75^,^76^,^77^. Asking participants to attend to *why* an agent is performing a certain action as compared to *how* the action is being performed elicits increased activity in l-aSTS^63^. The l-aSTS is also involved in representing the valence dimension of mental state representations, which captures the difference between positive and negative mental states^78^. While reasoning about agents acting with false (versus true) beliefs, l-aSTS shows significant percent signal change from baseline^62^,^79^ and also comes online as early as 200–300 ms^80^. This work indicates the involvement of l-aSTS in ToM^4^. But exactly which aspect of ToM does this region support? The umbrella term ToM in its broadest sense represents the capacity to process the representational mental states of other agents, but this process relies on other basic component processes as well (e.g., causal reasoning, self/other distinction, face recognition, gaze processing, etc.)^81^. Therefore, it has been argued that there is not one homogeneous ToM network or mentalizing system but rather different neural regions for distinct aspects of ToM^82^. Indeed, recent meta-analyses^64^,^65^ suggest heterogeneous functional profiles associated with different types of ToM tasks (e.g. false belief, social animations, strategic games, etc.). What specific aspect of ToM is the l-aSTS responsible for? Some prior work sheds light on this issue. A systematic study contrasting social animations versus false belief tasks revealed that the TPJ primarily supports predicting behavior based on covert mental states, while both aSTS and pSTS support decoding intentions or mental states from animate motion or perceived actions^61^, consistent as well with more exhaustive meta-analytic investigations^64^. According to these authors, the broader term “intention” belies the graded distinction between two types of mental states^83^: (*i*) “intention-in-action”: perceiving intentions and goals in the actions of agents (e.g., social animation tasks) (subserved by l-aSTS); (*ii*) “prior intention”: representing intentions based on covert mental states (e.g., false belief tasks) that may or may not lead to immediate action (subserved by TPJ). Indeed, some developmental evidence also supports this distinction: the representation of others’ intentions decoded from actions develops before the representation of covert mental states^84^.

According to this view, the role of l-aSTS during the moral judgment task is to infer the nature of the agent’s intent (malicious or innocent) based on the action the agent performed. Notably, in real life, we rarely have access to agents’ internal mental states; agents’ external actions are what we tend to rely on when considering their goals and intentions^85^,^86^. Thus, we speculate that the observed structure-behavior correlation represents participants’ dispositional tendency in daily life to infer the intentions underlying actions based on observation of actions rather than by explicit representation of mental state information. The greater this tendency, the more adept the participant will be in attributing reduced harmful intent to accidental harm-doers. The current result is also consistent with prior work showing activity in l-aSTS during forgivability judgments^67^.

To some, it may be surprising that we did not find the VBM effect at rTPJ, given the amount of research that places rTPJ at the center of morally relevant mental state reasoning^16^,^17^,^18^. We discuss this null effect at length in Supplementary Text S6.

### What do the current VBM results signify?

In the VBM analysis, whole-brain voxel-wise regression analyses were restricted to the functionally defined ToM network to investigate the link between individual differences in moral condemnation and variation in local GMV in the network. This analysis revealed one robust effect: more lenient moral judgments of accidental harms were correlated with greater GMV at l-aSTS. What does this result signify at the mechanistic level?

It is still unclear how and why individual differences in brain morphometry are found to be correlated with personality traits and task performance, but it is often assumed that greater GMV is associated with better computational efficiency of that region^48^, which in turn leads to enhanced task performance. Indeed, grey matter reduction in l-aSTS is associated with ToM deficits in schizophrenia patients^66^. Therefore, the current findings can be interpreted in the following way: individuals with greater GMV in l-aSTS tend to exculpate an agent who causes harm accidentally because they are better at generating a robust representation of an agent’s innocent intentions needed to compete with prepotent negative arousal elicited by harmful consequences, which would lead to condemnation^87^. In other words, greater GMV in l-aSTS enhances computational efficiency of generating and processing mental state representation, which in turn leads to greater reliance on this information for moral judgment.

This raises an even more interesting question as to why some people have greater GMV at aSTS than others to begin with. While a cross-sectional study like ours can’t arbitrate on this issue (or determine the causal direction of the relationship between brain structure and moral judgment), we offer some speculation here. On the one hand, it is known that individual differences in GMV at focal brain areas are highly heritable^88^,^89^. It is therefore possible that genes contribute to variation in GMV at l-aSTS and thus to variation in moral judgments (gene → structure → judgment). On the other hand, the alternative causal pathway is equally valid (environment → judgment → structure). Different environments (cultures, societies, etc.) differ in the *degree* to which they place emphasis on intent versus outcome for moral judgment^2^,^90^,^91^. These environmental influences are associated with variation in brain regions due to use-dependent brain plasticity such that the size of a brain region is influenced by its use (neurons that regularly “fire together, wire together”^92^). In addition to these two possibilities, much more complicated interplay can also be expected between genes and environment via either interaction and/or correlation^88^.

### Limitations

As a limitation, we note that although, traditionally, behavioral data for VBM studies are collected out of the scanner, this was not the case for the current study. Some recent work shows that participants are slower and exhibit poor focus on the task while in scanner as opposed to the lab environment, though this difference was observed for a perceptual decision-making task^93^ and its relevance for social decision-making remains unclear. Future studies can also explore how variety of other demographic details^94^ (Big Five personality traits, education, ethnicity, etc.) and more realistic contexts^95^ affect moral judgments rather than relying primarily on a uniform group university students and hypothetical text vignettes as sampled in the current study.

## Conclusion

In conclusion, we have shown here that the interindividual differences in the severity of moral judgments about unintentional harmful behaviors are associated with volumetric differences in the left aSTS, a region implicated in reasoning about others’ mental states, such that the greater the grey matter volume, the less accidental harm-doers are condemned.

## Additional Information

**How to cite this article**: Patil, I. *et al*. Neuroanatomical correlates of forgiving unintentional harms. *Sci. Rep.* **7**, 45967; doi: 10.1038/srep45967 (2017).

**Publisher's note:** Springer Nature remains neutral with regard to jurisdictional claims in published maps and institutional affiliations.

## Supplementary Material

Supplementary Information
