# Supplementary material for: Neuroanatomical correlates of forgiving unintentional harms
Source: Sci Rep. 2017 Apr 6;7:45967. doi: 10.1038/srep45967 (PMC5382676; doi:10.1038/srep45967)
Supplement: Supplementary Information [file srep45967-s1.pdf]

Supplementary Information for "Neuroanatomical correlates of forgiving unintentional harms"

Indrajeet Patil[1,2✉], Marta Calò[3*], Federico Fornasier[3*], Liane Young[4], Giorgia Silani[5]

[1]Scuola Internazionale Superiore di Studi Avanzati, Neuroscience Sector, Trieste, Italy.
[2]Department of Psychology, Harvard University, Cambridge, MA, USA.
[3]University of Trieste, Trieste, Italy.
[4]Department of Psychology, Boston College, Boston, USA.
[5]Department of Applied Psychology: Health, Development, Enhancement and Intervention, University of Vienna, Austria.

*These authors contributed equally to this work.

✉Correspondence should be addressed to:

Indrajeet Patil, 33 Kirkland Street, Cambridge, Massachusetts, 02138, USA. E-mail:

patilindrajeet.science@gmail.com

Abbreviations:

dmPFC = dorsomedial prefrontal cortex, gICA = group ICA, GLM = general linear model, GMV = grey matter volume, ICA = Independent component analysis, PC = precuneus, PI = posterior insula, ToM = theory of mind, TP = temporal pole, TPJ = temporoparietal junction, STS = superior temporal sulcus, SVC = small volume correction, VBM = voxel-based morphometry, vmPFC = ventromedial prefrontal cortex

Supplementary Text S1: Scenario details

Scenario type by version breakdown. Red and green cells denote scenarios taken from previous studies (Cushman, 2008; Young, Camprodon, Hauser, Pascual-Leone, & Saxe, 2010), resp.

No.	scenario	v1	v2	v3	v4
1	Popcorn	neu	att	int	acc
2	Malaria Pond/African pond	att	int	acc	neu
3	Spinach	int	acc	neu	att
4	Peanut allergy	acc	neu	att	int
5	Rabies/Rabid dog	neu	att	int	acc
6	Meatloaf	att	int	acc	neu
7	Seatbelt/Amusement park	int	acc	neu	att
8	Teenagers/Skiing	acc	neu	att	int
9	Ham sandwich	neu	att	int	acc
10	Safety Cord/Rock climbing	att	int	acc	neu
11	Sesame seeds	int	acc	neu	att
12	Coffee/Chemical Plant	acc	neu	att	int
13	Bridge	neu	att	int	acc
14	Pool	att	int	acc	neu
15	Mushrooms	int	acc	neu	att
16	Latex	acc	neu	att	int
17	Motorboat	neu	att	int	acc
18	Asthma	att	int	acc	neu
19	Veterinarian/Dog poison	int	acc	neu	att
20	Zoo	acc	neu	att	int
21	Sushi	neu	att	int	acc
22	Cayo/Monkeys	att	int	acc	neu
23	Wet floor	int	acc	neu	att
24	Lab	acc	neu	att	int
25	Vitamin	neu	att	int	acc
26	Airport	att	int	acc	neu
27	Chairlift	int	acc	neu	att
28	Bike	acc	neu	att	int
29	Safety Town/Fire drill	neu	att	int	acc
30	Parachute	att	int	acc	neu
31	Sculpture	int	acc	neu	att
32	Dentist	acc	neu	att	int
33	Iron	neu	att	int	acc
34	Tree House	att	int	acc	neu
35	Jellyfish/Ocean	int	acc	neu	att
36	Laptop	acc	neu	att	int

Note: The exact wording of the details can be found in the original papers or can be requested from the corresponding author. Italian translations are also available on request.

Supplementary Text S2: Component corresponding to the ToM network in gICA analysis

Why gICA was preferred over GLM for analyzing ToM task data?

For 7 out of 49 participants, MATLAB randomization for this task failed and the stimuli for each condition were thus shown in consecutive manner. This effectively turned our event-related design into a blocked-design. Additionally, since the average length of individual stimulus was 35 s, the length of each block was roughly 160s (including ITI). This made our design highly inefficient to find any task-related signal in the General Linear Model (GLM) focusing on ToM > control videos contrast. This is because of the dominant low-frequency noise, which overshadows signal in such long blocked-designs (optimal length: 16-50 s; Henson, 2007). Thus, to boost statistical power by utilizing the entirety of sample, gICA (n = 49) was preferred over the standard GLM-based (n = 42) analysis.

Brief summary of rationale behind gICA

The ICA was preferred for localizing functional network over other approaches (e.g., seed-based correlation analysis) because it provides many advantages over other univariate approaches to functional connectivity in terms of accounting for artefactual influence of confounding signals, such as respiratory, cardiovascular, non-grey matter, etc. (Cole, Smith, & Beckmann, 2010). The group-ICA method, as implemented in GIFT, involves following steps: initially data from all subjects are spatially normalized and dimensionally reduced by conducting PCA at individual subject level. All reduced datasets are then temporally concatenated to form one dataset on which group-ICA is applied. When applied, group-ICA decomposes a two-dimensional data matrix (with columns representing time course of voxels and rows representing different subjects) into two matrices, one corresponding to the time courses of components for the group and the other corresponding to spatial maps for components with component loading for each voxel. Individual-level components are then created via GICA back-reconstruction method based on PCA compression and projection.

Identifying the ToM component

We closely followed the analysis protocol detailed in a previous study (Hyatt, Calhoun, Pearlson, & Assaf, 2015) and we provide here extensive details about the preprocessing pipeline as data preprocessing can affect gICA results (Vergara, Mayer, Damaraju, Hutchison, & Calhoun, 2016). Subject-specific principal component analysis (PCA) was run to retain 30 principal components (PCs). At the second stage of group data reduction, expectation maximization algorithm was used to retain a final set of 20 PCs (a low model order ICA, i.e.). The Infomax algorithm was then run, repeating it 15 times in ICASSO, to generate a stable set of final 20 components. Finally, subject-specific spatial maps (SMs) and time courses (TCs) were estimated using the GICA back-reconstruction method based on PCA compression and projection. Before looking for the component that represented the ToM network, we first identified a subset of components that were considered BOLD-related brain networks rather than physiological artifacts. To this effect, component viewer facility in GIFT was utilized to compute the frequency spectrum of each component TC and the dynamic range (DR) and fractional amplitude of low-frequency fluctuation (fALFF). Based on frequency-domain analysis (Allen et al., 2011), it has been shown that artefactual components often exhibit both low DR and low fALFF and such components were removed from further analysis after visual inspection (Griffanti et al., 2016). Based on this analysis, 6 components were removed. Out of the 14 biologically meaningful and non-artefactual remaining components, the component corresponding to the ToM network was identified by using spatial correlation feature within GIFT, which identified the component (shown in figure below) with the highest spatial correspondence (correlation: r = 0.4152; multiple regression: β = 0.1567) to the ToM meta-analytic functional map (Mar, 2011). Redoing the same step with a different meta-analytic functional map for ToM studies (Bzdok et al., 2012) also revealed identical component (correlation: r = 0.3135; multiple regression: β = 0.0983).

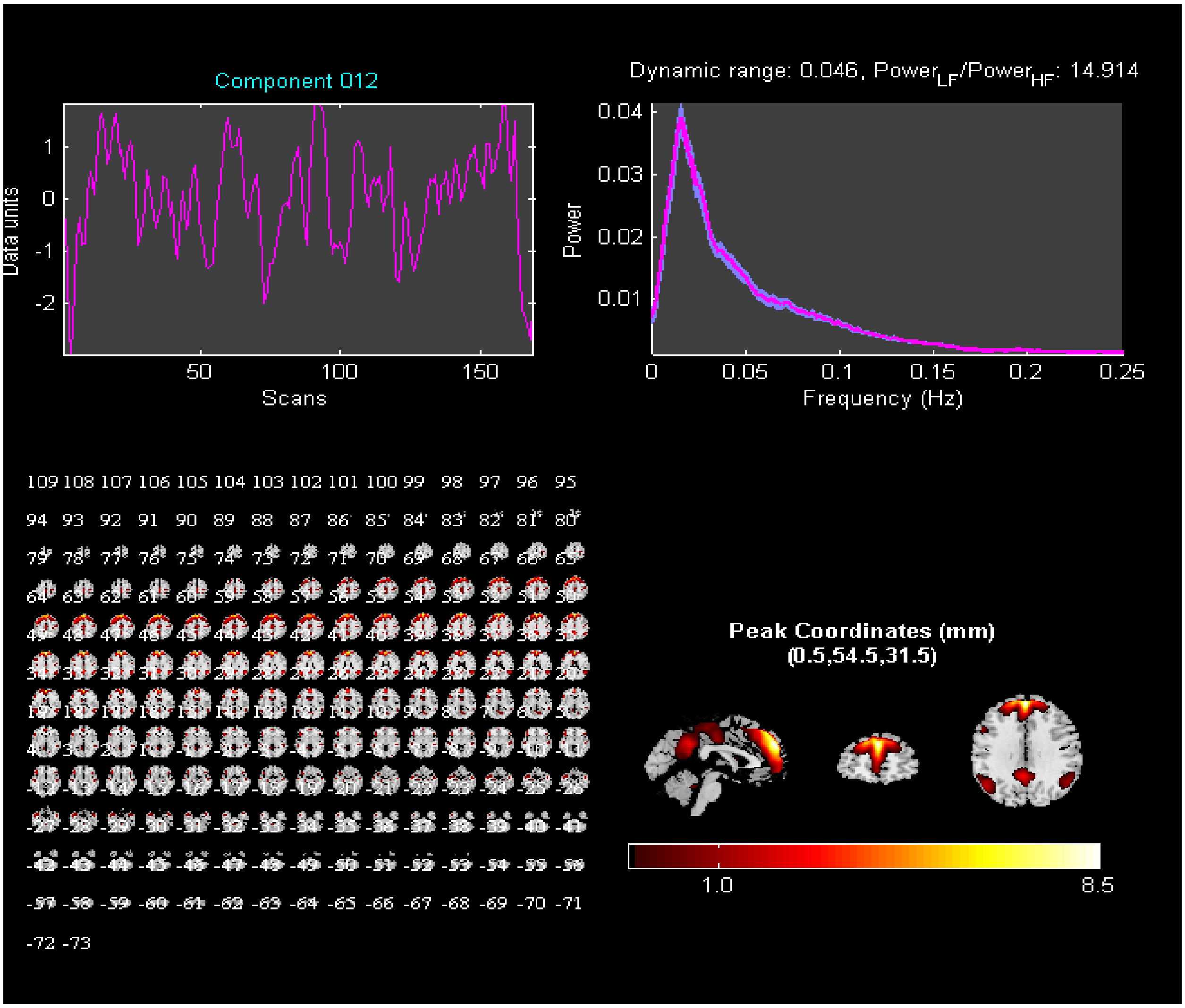

Coordinates for the ToM component

Significant clusters of activation from the component corresponding to the ToM network[1] and anatomical labels derived using Anatomy toolbox (Eickhoff et al., 2005) are provided in the table below.

[1] In passing, we note that our findings contest the claim made by a previous study that mPFC is not necessary for mentalizing (Otti, Wohlschlaeger, & Noll-Hussong, 2015). This study utilized the same task as we did, but did not find any activation in mPFC. Importantly, the study had only 20 participants. In the current study, we observed a robust activation in the mPFC both in model-free (n = 49) and model-based (n = 42) analysis (as did Moessnang et al., 2016). Thus, we maintain that "absence of evidence was not evidence of absence", i.e., study by Otti and colleagues failed to reject the null hypothesis in mPFC due to low statistical power stemming from their small sample size.

Region	MNI Coordinates (in mm) x	y	z	Cluster-level *p*-value (FWE-corr)	Cluster size *k* (voxels)	Peak voxel *p*-value	Peak voxel *t*-score
(R Superior Medial Gyrus)	2	54	28	< 0.0001	7678	< 0.0001	23.1337
(L Superior Medial Gyrus)	0	48	36			< 0.0001	19.4130
(L Superior Frontal Gyrus)	-16	38	46			< 0.0001	18.5216
(L PCC)	-4	-54	32	< 0.0001	2626	< 0.0001	15.7288
(L MCC)	-4	-18	40			< 0.0001	10.9699
(L Calcarine Gyrus)	-2	-62	14			< 0.0001	9.9628
(L Middle Temporal Gyrus)	-48	-62	22	< 0.0001	1269	< 0.0001	14.2608
(L Angular Gyrus)	-48	-66	30			< 0.0001	12.3867
(L Angular Gyrus)	-44	-54	26			< 0.0001	12.1590
(L IFG (p. Triangularis))	-52	22	4	< 0.0001	1351	< 0.0001	12.6406
(L Middle Temporal Gyrus)	-52	-6	-18			< 0.0001	12.1858
(L IFG (p. Orbitalis))	-42	28	-10			< 0.0001	11.6126
(R Angular Gyrus)	52	-60	24	< 0.0001	846	< 0.0001	12.5966
(R Angular Gyrus)	48	-48	28			< 0.0001	8.6377
(R Middle Temporal Gyrus)	52	-16	-14	< 0.0001	411	< 0.0001	12.0895
(R Middle Temporal Gyrus)	62	-16	-10			< 0.0001	9.2006
(R Middle Temporal Gyrus)	54	-6	-20			< 0.0001	8.9386
(RPrecentral Gyrus)	46	-14	54	< 0.0001	829	< 0.0001	11.4522
(R Postcentral Gyrus)	28	-28	56			< 0.0001	9.2680
(RPrecentral Gyrus)	52	-12	44			< 0.0001	9.2596
(R Postcentral Gyrus)	28	-38	58	< 0.0001	161	< 0.0001	10.2332
(L Postcentral Gyrus)	-40	-20	42	< 0.0001	408	< 0.0001	9.7387
(L Postcentral Gyrus)	-50	-14	44			< 0.0001	9.3724
(L Postcentral Gyrus)	-50	-12	34			< 0.0001	7.9858
(L Middle Temporal Gyrus)	-54	-36	-2	< 0.0001	119	< 0.0001	9.3668
(R IFG (p. Orbitalis))	42	28	-14	< 0.0001	99	< 0.0001	9.2627
(L Caudate Nucleus)	-12	12	14	< 0.0001	123	< 0.0001	8.5429
	-26	-26	52	< 0.0001	156	< 0.0001	8.3613
(L Paracentral Lobule)	-14	-32	64			0.0012	6.7847
(R Caudate Nucleus)	12	6	18	< 0.0001	92	< 0.0001	8.0743
(R Caudate Nucleus)	10	14	12			0.0001	7.4071
(L Hippocampus)	-22	-14	-20	< 0.0001	65	0.0001	7.6082
(L Hippocampus)	-28	-10	-24			0.0007	6.9398
(R Middle Occipital Gyrus)	38	-84	8	< 0.0001	46	0.0001	7.4523
(R Middle Occipital Gyrus)	30	-86	16			0.0045	6.4181
(R Superior Parietal Lobule)	24	-50	62	< 0.0001	22	0.0001	7.4500
(L Middle Temporal Gyrus)	-58	-46	4	< 0.0001	26	0.0013	6.7810
(R Lingual Gyrus)	14	-50	4	0.0001	17	0.0016	6.7164
(R Cerebelum (IV-V))	8	-52	-2			0.0018	6.6784
(R Middle Frontal Gyrus)	42	20	46	< 0.0001	26	0.0042	6.4418
(R Medial Temporal Pole)	46	16	-32	0.0004	12	0.0046	6.4161

Supplementary Text S3: Additional details about VBM analysis

For the sake of brevity, we provide some additional details about the preprocessing of the anatomical data and GLM modeling choices made during the VBM analysis.

- All images were inspected for the common scanner artifacts by the authors (with the help of following references: Graves & Mitchell, 2013; Stadler, Schima, Ba-Ssalamah, Kettenbach, & Eisenhuber, 2007) and for structural abnormalities by in-house physicians and technicians.
- The same analysis software (SPM) and operating system (Windows) were used across all subjects, since this can affect morphometry estimates (Chepkoech, Walhovd, Grydeland, & Fjell, 2016; Glatard et al., 2015; Gronenschild et al., 2012).
- During segmentation routine, intensity distributions for each tissue class was modeled using numerous Gaussians: two for GM, two for WM, two for CSF, three for bone, four for other soft tissues, and two for air (background).
- The images in native space were used for calculating total intracranial volume (TIV; by summing tissue volume for GM, WM, and CSF) with the help of Tissue Volumes Utility of SPM12, which has been shown to be a highly reliable way to compute TIV in both control and clinical populations (Malone et al., 2015; Ridgway, Barnes, Pepple, & Fox, 2011; Sargolzaei et al., 2015).
- Normalization was carried out using DARTEL toolbox because it has been shown to be a more sensitive approach to morphometry analyses than the standard and optimized VBM (Li et al., 2013). Note that since DARTEL, as implemented here, was used to create a study specific group template, it is important to note that characteristic of the group can affect the final individual normalized tissue maps (Michael, Evans, & Moore, 2016). This can create artefactual group differences, but since the current study did not feature such comparison it is immune to this criticism.

- Spatial smoothing was applied normalized GM in DARTEL space in order to - (*i*) validate choice of parametric tests, (*ii*) account for residual individual differences remaining from normalization (cf. Bookstein, 2001), and (*iii*) increase signal-to-noise ratio (Kurth, Luders, & Gaser, 2015)
- Quality assurance review of the final smoothed GM images was performed using VBM8 toolbox. Sample homogeneity was assessed using a covariance matrix and volumes with an overall covariance below two standard deviations were inspected further to ensure that there were no abnormalities in these volumes.
- For the regression models, none of the continuous covariates was mean centered as the contrasts testing for the average effect were irrelevant for the VBM analysis (cf. http://mumford.fmripower.org/mean_centering/).
- Global normalization was achieved by entering TIV values as globals (as recommended by Ridgway et al., 2009) in proportional scaling. This means that GM volume at each voxel was first divided by TIV and statistical analysis was carried out on these values. Note that proportional scaling significantly changes the interpretation of the final results (cf. Mechelli, Price, Friston, & Ashburner, 2005 and http://en.wikibooks.org/wiki/SPM/VBM). Modulated images with no globals applied reveal brain regions with *absolute* volumetric differences in GM, while modulated images with TIVs as globals reveal brain regions containing disproportionately larger or smaller (compared to TIV) amount of GM. We acknowledge that although the proportional relation between GM volume and TIV doesn't hold when TIV is the only regressor in the model (for more, see Liu, Johnson, Long, Magnotta, & Paulsen, 2014), it can't be rejected when age and gender are additionally included in the regression model (Barnes et al., 2010), as we have done here. We additionally checked whether the current data met the assumptions of the proportional scaling method to ensure validity of this approach of adjustment for brain size over other approaches (ANCOVA, residual method, etc.; for more, see O'Brien et al., 2011).

- To avoid activations lying outside of the brain (due to low variance problem; Ridgway, Litvak, Flandin, Friston, & Penny, 2012) and to increase power of FWE-correction by reducing analysis regions, we created a mask using the Masking Toolbox (Ridgway et al., 2009; http://www0.cs.ucl.ac.uk/staff/g.ridgway/masking/), which attempts to find an optimal threshold to binarize an average (GM) image based on correlation with the average image. Any voxel that fell outside of this mask was excluded from the analysis. This has been shown to be a more reliable approach (in terms of likelihood of false negatives) than using an arbitrary threshold (e.g., 0.2) to remove voxels with intensity below this value (Ridgway et al., 2009).
- All statistical parametric maps are displayed on smoothed, representative scans (average of 305 T1 images, provided in SPM12; Ridgway et al., 2008). All peaks of activations are reported in MNI-coordinates but no Brodmann Area (BA) labels have been reported as assigning functional activations to cytoarchitectotonically defined BAs can be inaccurate in the absence of probabilistic maps of underlying cytoarchitectotonic variability (Devlin & Poldrack, 2007).
- Recent work has begun to reveal that within motion scanner of even few mm per minute can severely affect morphometric estimates of GM (Alexander-Bloch et al., 2016), and, although prospective motion correction techniques have been introduced to account for such effects (Stucht et al., 2015), no such correction was implemented in the current study due to lack of necessary equipment (Maclaren, Herbst, Speck, & Zaitsev, 2013).

  Additionally, time-of-day can affect all major tissue classes (GM, WM, CSF) such that apparent brain volume reduces from morning to evening (Nakamura, Brown, Narayanan, Collins, & Arnold, 2015), and this has greater impact on morphometric measures of frontal and temporal lobes (Trefler et al., 2016). Although we did not explicitly account for this effect, all participants were scanned during relatively fixed hours in the afternoon (15:00 to 19:00).

  Thus, we acknowledge that in-scanner movement and time-of-day may have contributed to noise in our data, but we find a systematic biasing of our results to be unlikely.

- We note here that although we computed GMV using volume-based representation, the GMV computed using surface-based representation tends to be highly correlated with this method (Winkler et al., 2010). Additionally, it is interesting to note that variation in surface area and cortical thickness are two independent contributors to variation in GMV at both regional and local level, with surface area being the more significant contributor (Winkler et al., 2010). Thus, future studies can use surface-based morphometry techniques to investigate the same question. We would predict that a similar effect would be observed in l-aSTS: greater surface area would be associated with reduced moral condemnation for accidents.
- *Why should we carry out VBM analysis?*[2]

  One may wonder as to why we need to carry out VBM analysis (or any other morphometry studies in general) when functional neuroimaging studies can inform us about functional correlates of inter-individual differences in behavior. There are various advantages afforded by VBM analysis that make it a complementary approach to assess the source of individual differences (Kanai & Rees, 2011)-

  (*i*) Inter-individual variability in human behavior can be predicted from the structure of the human brain (grey matter volume, cortical thickness, surface area, white matter tracts, etc.) measured with MRI. Although the same holds true for functional neuroimaging, the advantage provided by VBM is that they allow experimenters to link an individual's performance measured in an ecologically valid environment to brain structure measurement. Such assessment can be difficult to implement in MRI scanner settings in certain tasks, for example tasks that require participant to interact with multiple other individuals simultaneously, and thus can't be studied in an fMRI study (Camerer & Mobbs, 2017).

  (*ii*) Since the heritability of a particular cognitive function (e.g., mental state reasoning) is contingent on the extent to which the respective brain structures (e.g., rTPJ) are influenced by genetic factors (Ge et al., 2016; Winkler et al., 2010), morphometry studies can be used to

[2] We were prompted to discuss this based on a reviewer suggestion.

generate interesting hypotheses for multimodal studies linking function, structure, genes, and behavior.

(*iii*) Correlational data between GMV and performance can also be used to generate interesting hypotheses as to which brain regions might be important for the performance of the task. For example, in the current study, it was only *after* we found VBM effect at aSTS that we decided to carry out new analysis on our fMRI data to see if there was any correlation between functional activity in this region and performance. A further prediction can also be made for conducting neurostimulation studies (TMS, tDCS) whereby disrupting the function of aSTS (mental state reasoning) would lead to reduced task performance (increased condemnation of accidental harm cases).

Supplementary Text S4: Replicating the results with the GLM-based ToM mask

In this section, we show that even if we focus only on these 42 participants, we still get all primary regions of interest observed with gICA and also get VBM effects in the same region.

Replicating primary VBM results in the GLM-derived ToM mask

Here we focused only on the 42 participants for whom the MATLAB randomization did not fail and thus the General Linear Model (GLM) approach was appropriate for modelling the data (see main text). The ToM network at group-level was localized by entering beta-weights from canonical HRF contrasts from first-level in a one sample t-test. Whole-brain analyses were thresholded at $p < 0.05$, Family-wise Error (FWE) corrected at the threshold level (cluster-defining threshold: $p < 0.05$ (corrected), extent threshold: $k > 10$). The results were masked with meta-analysis map from (Mar, 2011). The results revealed the expected nodes of the ToM network, viz. bilateral temporoparietal junction (TPJ), sections of medial prefrontal cortex (mPFC), temporal poles (TP), superior temporal sulcus (STS), and precuneus (PC).

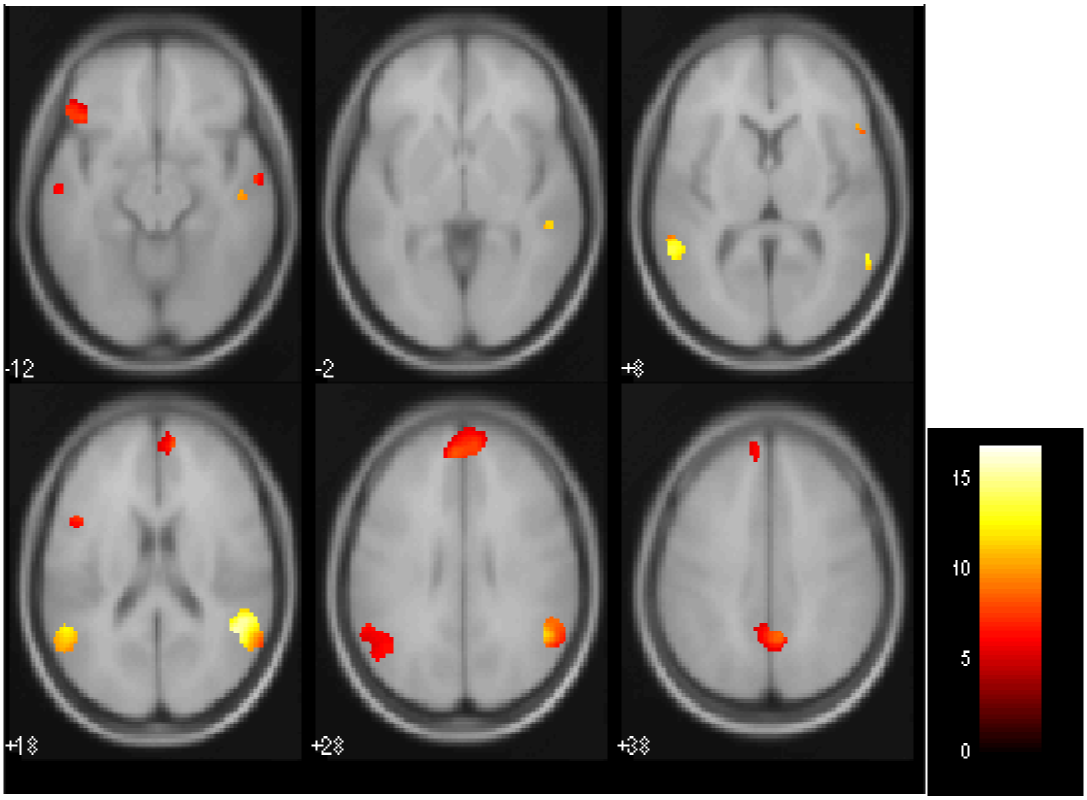

Note: The accompanying color bar in the figure below denotes *t*-values.

List of all coordinates along with labels derived from the Anatomy toolbox (Eickhoff et al., 2005) is provided below:

MNI coordinate			*p* (FWE-corrected)	*k*	Label (Anatomy toolbox)
x	*y*	*z*			
50	-42	10	< 0.0001	730	(R Middle Temporal Gyrus)
46	-52	20			(R Middle Temporal Gyrus)
-56	-50	10	< 0.0001	583	(L Middle Temporal Gyrus)
52	28	4	0.0120	24	(R IFG (p. Triangularis))
52	-38	2	0.0058	33	(R Middle Temporal Gyrus)
54	2	-20	< 0.0001	238	(R Middle Temporal Gyrus)
48	-18	-12			
54	20	14	0.0092	28	(R IFG (p. Opercularis))
4	-54	44	< 0.0001	262	(R Precuneus)
-6	48	32	< 0.0001	627	(L Superior Medial Gyrus)
10	54	20			(R Superior Medial Gyrus)
-46	28	-6	0.0006	156	(L IFG (p. Orbitalis))
-46	14	20	0.0024	34	(L IFG (p. Opercularis))
-52	0	-22	0.0086	46	(L Middle Temporal Gyrus)
26	-2	-20	0.0108	19	(R Amygdala)
36	22	-22	0.0136	32	(R IFG (p. Orbitalis))
-56	-16	-12	0.0105	23	(L Middle Temporal Gyrus)
-26	0	-22	0.0141	24	(L Amygdala)

Same multiple regression models used in the main text to explore GMV and moral condemnation association on voxel-level were used, but with one crucial difference: for image-based small volume correction the ToM mask used was from the GLM-analyzed localizer data instead of gICA-analysed.

The results again revealed that more severe moral condemnation for accidental harm condition was associated with reduced GMV in the left aSTS/MTG (x = -60, y = -12, z = -14; β = -0.0268; k = 17; p(FWE-corrected) = 0.0116). No such activation was found in the right aSTS/MTG. Additionally, no such result was found for any of the other conditions. Thus, the same result is obtained irrespective of whether we used a functional mask derived from GLM-based analysis of the ToM localizer task or gICA-based.

Supplementary Text S5: Descriptive statistics for moral condemnation

Average for condition	Range	Min	Max	Mean	SD
neutral	4.06	1.06	5.12	2.12	0.90
accidental	4.81	1.67	6.47	4.11	1.16
attempted	5.17	1.67	6.83	5.19	1.03
intentional	2.50	4.50	7.00	6.34	0.62

Supplementary Text S6: Exploratory analysis at rTPJ

VBM results at rTPJ: Although no effect was observed for any condition between the grey matter volume in rTPJ with moral judgment in any condition even at a more liberal, uncorrected threshold of $p < 0.001$. Below is a scatter plot illustrating the negative linear associations between GMV in rTPJ ($\rho(47) = -0.316$, $p = 0.027$, $n = 49$, two-tailed) and the severity of moral condemnation of accidental harm at a highly liberal threshold ($p < 0.05$ (uncorrected)) for a comparison with Figure 2 in the main text. The solid lines indicate a linear fit to the data, while the curved lines represent mean 95% confidence intervals for these lines. Extracted grey matter volume data presented in figures are non-independent of the statistical test conducted and should *not* be used for effect-size estimates (Vul & Pashler, 2017). They are included here only as a visual aid for interpretation of results.

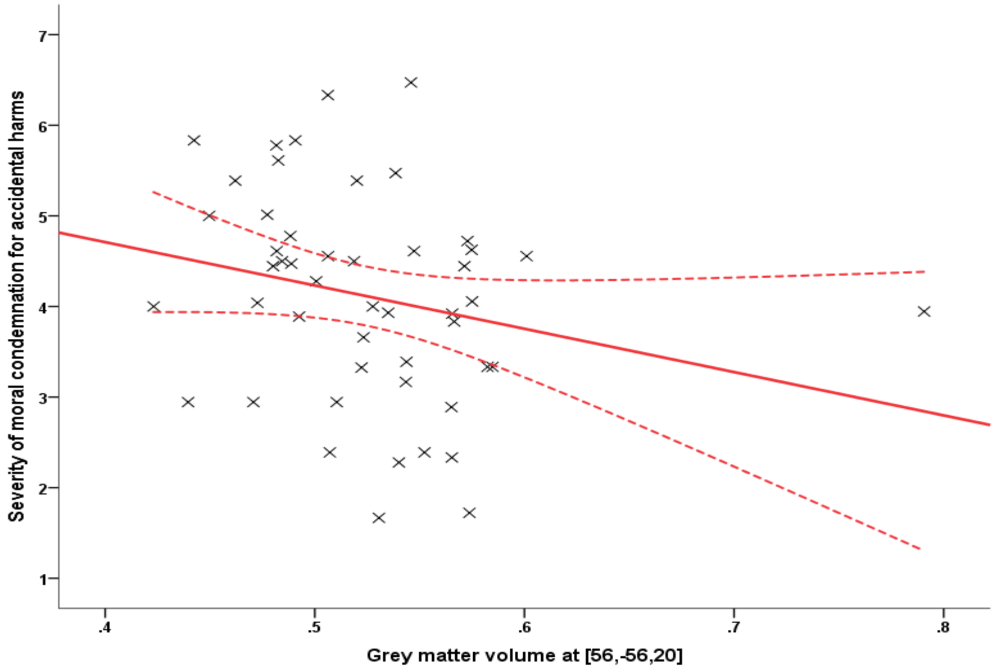

fMRI results at rTPJ: For ROI analysis, the data from spherical ROIs with a radius of 8 mm was extracted from rTPJ at [56, -56, 20] in MarsBar. This analysis revealed that there was indeed a negative correlation between parameter estimate during *acceptability* segment and reduced condemnation for accidents, but this effect was not significant ($\rho(40) = -0.213$, $p = 0.088$, $n = 42$, one-tailed).

Discussion: *Why the predicted effect was not observed in the rTPJ?*

As noted in the main text, we did not find any effect at the rTPJ[3], which is surprising given the amount of research that places rTPJ at the center of morally relevant mental state reasoning (reviewed in Chakroff & Young, 2015). As argued in the main text, this can be due to the different aspects of ToM subserved by different regions (l-aSTS versus rTPJ) such that the aspect relevant for shaping the brain-structure relationship is neurally grounded in l-aSTS. Nevertheless, we entertain other mutually compatible explanations for this null effect below.

One plausible explanation is that the underlying assumption about equivalence between structural and functional relationship is misplaced when it comes to mental state attribution. We would expect the VBM effect at rTPJ based on a previous functional data (Chakroff et al., 2016; Young & Saxe, 2009). Functional imaging studies deal with short-lived brain-behavior association while structural imaging deal with long-term brain-behavior associations and it is possible that for some regions there is no homologous

[3]It is noteworthy that morphometry studies exploring neuroanatomical correlates of ToM skills have found a wide variety of regions, some usual suspects, like TPJ and dmPFC (Rice & Redcay, 2015; Valk et al., 2017), while others that are not as extensively discussed in the ToM literature, e.g., anterior temporal lobes (Irish, Hodges, & Piguet, 2014), right inferior frontal gyrus (Rice & Redcay, 2015), ventrolateral prefrontal cortex (Hirao et al., 2008), amygdala (Rice, Viscomi, & Riggins, 2014), middle temporal gyrus (Grosse Wiesmann, Schreiber, Singer, Steinbeis, & Friederici, 2017), etc. Thus, although much more attention is paid to certain regions of the ToM network (TPJ, PC, etc.), GMV of other regions that are consistently found across studies and paradigms are also important in explaining inter-individual variation in ToM skills.

mapping of functional and structural variation, i.e., functional activation differences may (aSTS) or may not (rTPJ) be reflected in structural differences (and vice versa), at least at the macroscopic level (Kanai & Rees, 2011; Lewis, Kanai, Bates, & Rees, 2012). An important and unresolved issue in VBM research is determining which functions are linked to structural differences (Kanai & Rees, 2011); a systematic and principled investigation is in order.

Another possible explanation involves our choice of ToM functional localizer task. Note that while the localizer task featuring social animations recruits spontaneous mental state attribution to interacting triangles, the moral judgment task required false belief reasoning. Thus, there may have been a mismatch between the assumed functional role of the rTPJ across the two tasks. Indeed, our choice of localizer task was based on the tacit assumption of structurally invariant functional properties in the rTPJ across different types of tasks. Recent meta-analyses (Molenberghs, Johnson, Henry, & Mattingley, 2016; Schurz, Radua, Aichhorn, Richlan, & Perner, 2014), however, have argued against such context-insensitive specialization in the rTPJ. In particular, false belief tasks were found to elicit activity in the posterior parts of the rTPJ, while social animation tasks in the the anterior parts. Thus, future studies investigating the neuroanatomical correlates of a specific cognitive process should pay close attention to the choice of functional localizer task based on the task of primary interest.

Yet another possibility is that no effect at aSTS was found in previous functional studies because most of the prior investigations have focused on few ROIs (dmPFC, vmPFC, bilateral TPJ, PC), while ignoring the rest of the ToM network (cf. Chakroff et al., 2016; Koster-Hale, Saxe, Dungan, & Young, 2013; Young & Saxe, 2008, 2009). The current investigation was unbiased in this sense, since we interrogated the entire ToM network localized using gICA and did not focus only on the few key nodes of the network.

In passing, we also note that previous morphometry studies examining how regional variation in brain structure relates to individual differences in endorsed moral values (Lewis et al., 2012), moral reasoning skills (Prehn et al., 2015), prosocial behavior (Marsh et al., 2014; Thijssen et al., 2015; Yamagishi et al., 2016), and moral judgments in clinical populations (Baez et al., 2015, 2016) have not found any

correlations with GMV or cortical thickness of TPJ region. To our knowledge, only study has found any effect in this region: a positive correlation between rTPJ and altruistic decision making (Morishima, Schunk, Bruhin, Ruff, & Fehr, 2012).

References

Alexander-Bloch, A., Clasen, L., Stockman, M., Ronan, L., Lalonde, F., Giedd, J., & Raznahan, A. (2016). Subtle in-scanner motion biases automated measurement of brain anatomy from in vivo MRI. *Human Brain Mapping*, *37*(7), 2385–2397. http://doi.org/10.1002/hbm.23180

Allen, E. A., Erhardt, E. B., Damaraju, E., Gruner, W., Segall, J. M., Silva, R. F., … Calhoun, V. D. (2011). A baseline for the multivariate comparison of resting-state networks. *Frontiers in Systems Neuroscience*, *5*, 2. http://doi.org/10.3389/fnsys.2011.00002

Baez, S., Kanske, P., Matallana, D., Montañes, P., Reyes, P., Slachevsky, A., … Ibanez, A. (2016). Integration of Intention and Outcome for Moral Judgment in Frontotemporal Dementia: Brain Structural Signatures. *Neuro-Degenerative Diseases*, *16*(3–4), 206–17. http://doi.org/10.1159/000441918

Baez, S., Morales, J. P., Slachevsky, A., Torralva, T., Matus, C., Manes, F., & Ibanez, A. (2015). Orbitofrontal and limbic signatures of empathic concern and intentional harm in the behavioral variant frontotemporal dementia. *Cortex*, *75*, 20–32. http://doi.org/10.1016/j.cortex.2015.11.007

Barnes, J., Ridgway, G. R., Bartlett, J., Henley, S. M. D., Lehmann, M., Hobbs, N., … Fox, N. C. (2010). Head size, age and gender adjustment in MRI studies: A necessary nuisance? *NeuroImage*, *53*(4), 1244–1255. http://doi.org/10.1016/j.neuroimage.2010.06.025

Bookstein, F. L. (2001). “Voxel-based morphometry” should not be used with imperfectly registered images. *NeuroImage*, *14*(6), 1454–1462. http://doi.org/10.1006/nimg.2001.0770

Bzdok, D., Schilbach, L., Vogeley, K., Schneider, K., Laird, A. R., Langner, R., & Eickhoff, S. B. (2012). Parsing the neural correlates of moral cognition: ALE meta-analysis on morality, theory of mind, and empathy. *Brain Structure & Function*, *217*(4), 783–96. http://doi.org/10.1007/s00429-012-0380-y

Camerer, C., & Mobbs, D. (2017). Differences in Behavior and Brain Activity during Hypothetical and Real Choices. *Trends in Cognitive Sciences*, *21*(1), 46–56. http://doi.org/10.1016/j.tics.2016.11.001

Chakroff, A., Dungan, J., Koster-Hale, J., Brown, A., Saxe, R., & Young, L. (2016). When minds matter for moral judgment: intent information is neurally encoded for harmful but not impure acts. *Social Cognitive and Affective Neuroscience*, *11*(3), 476–484. http://doi.org/10.1093/scan/nsv131

Chakroff, A., & Young, L. (2015). How the Mind Matters for Morality. *AJOB Neuroscience*, *6*(3), 43–48. http://doi.org/10.1080/21507740.2015.1058866

Chepkoech, J.-L., Walhovd, K. B., Grydeland, H., & Fjell, A. M. (2016). Effects of change in FreeSurfer version on classification accuracy of patients with Alzheimer’s disease and mild cognitive impairment. *Human Brain Mapping*, *37*(5), 1831–1841. http://doi.org/10.1002/hbm.23139

Cole, D. M., Smith, S. M., & Beckmann, C. F. (2010). Advances and pitfalls in the analysis and interpretation of resting-state FMRI data. *Frontiers in Systems Neuroscience*, *4*, 8. http://doi.org/10.3389/fnsys.2010.00008

Cushman, F. (2008). Crime and punishment: distinguishing the roles of causal and intentional analyses in moral judgment. *Cognition*, *108*(2), 353–80. http://doi.org/10.1016/j.cognition.2008.03.006

Eickhoff, S. B., Stephan, K. E., Mohlberg, H., Grefkes, C., Fink, G. R., Amunts, K., & Zilles, K. (2005). A new SPM toolbox for combining probabilistic cytoarchitectonic maps and functional imaging data. *NeuroImage*, *25*(4), 1325–1335. http://doi.org/10.1016/j.neuroimage.2004.12.034

Ge, T., Reuter, M., Winkler, A. M., Holmes, A. J., Lee, P. H., Tirrell, L. S., … Dice, L. R. (2016). Multidimensional heritability analysis of neuroanatomical shape. *Nature Communications*, *7*, 13291. http://doi.org/10.1038/ncomms13291

Glatard, T., Lewis, L. B., Ferreira da Silva, R., Adalat, R., Beck, N., Lepage, C., … Evans, A. C. (2015). Reproducibility of neuroimaging analyses across operating systems. *Frontiers in Neuroinformatics*, *9*, 12. http://doi.org/10.3389/fninf.2015.00012

Graves, M. J., & Mitchell, D. G. (2013). Body MRI artifacts in clinical practice: a physicist's and radiologist's perspective. *Journal of Magnetic Resonance Imaging*, *38*(2), 269–87. http://doi.org/10.1002/jmri.24288

Griffanti, L., Douaud, G., Bijsterbosh, J., Evangelisti, S., Alfaro-Almagro, F., Glasser, M. F., … Smith, S. M. (2016). Hand classification of fMRI ICA noise components. *NeuroImage*. http://doi.org/10.1016/j.neuroimage.2016.12.036

Gronenschild, E. H. B. M., Habets, P., Jacobs, H. I. L., Mengelers, R., Rozendaal, N., van Os, J., … Pappu, V. (2012). The Effects of FreeSurfer Version, Workstation Type, and Macintosh Operating System Version on Anatomical Volume and Cortical Thickness Measurements. *PLoS ONE*, *7*(6), e38234. http://doi.org/10.1371/journal.pone.0038234

Grosse Wiesmann, C., Schreiber, J., Singer, T., Steinbeis, N., & Friederici, A. D. (2017). White matter maturation is associated with the emergence of Theory of Mind in early childhood. *Nature Communications*, *8*, 14692. http://doi.org/10.1038/ncomms14692

Henson, R. (2007). Efficient experimental design for fMRI. In K. Friston, J. Ashburner, S. Kiebel, T. Nichols, & W. Penny (Eds.), *Statistical Parametric Mapping: The Analysis of Functional Brain Images* (pp. 193–210). London: Elsevier.

Hirao, K., Miyata, J., Fujiwara, H., Yamada, M., Namiki, C., Shimizu, M., … Murai, T. (2008). Theory of mind and frontal lobe pathology in schizophrenia: a voxel-based morphometry study. *Schizophrenia Research*, *105*(1–3), 165–74. http://doi.org/10.1016/j.schres.2008.07.021

Hyatt, C. J., Calhoun, V. D., Pearlson, G. D., & Assaf, M. (2015). Specific default mode subnetworks support mentalizing as revealed through opposing network recruitment by social and semantic FMRI tasks. *Human Brain Mapping*, *36*(8), 3047–63. http://doi.org/10.1002/hbm.22827

Irish, M., Hodges, J. R., & Piguet, O. (2014). Right anterior temporal lobe dysfunction underlies theory of mind impairments in semantic dementia. *Brain*, *137*(Pt 4), 1241–53. http://doi.org/10.1093/brain/awu003

Kanai, R., & Rees, G. (2011). The structural basis of inter-individual differences in human behaviour and cognition. *Nature Reviews Neuroscience*, *12*(4), 231–242. http://doi.org/10.1038/nrn3000

Koster-Hale, J., Saxe, R., Dungan, J., & Young, L. (2013). Decoding moral judgments from neural representations of intentions. *Proceedings of the National Academy of Sciences*, *110*(14), 5648–53. http://doi.org/10.1073/pnas.1207992110

Kurth, F., Luders, E., & Gaser, C. (2015). Voxel-Based Morphometry. In A. W. Toga (Ed.), *Brain*

Mapping: An Encyclopedic Reference (pp. 345–349). Academic Press: Elsevier. http://doi.org/10.1016/B978-0-12-397025-1.00304-3

Lewis, G. J., Kanai, R., Bates, T. C., & Rees, G. (2012). Moral values are associated with individual differences in regional brain volume. *Journal of Cognitive Neuroscience*, *24*(8), 1657–63. http://doi.org/10.1162/jocn_a_00239

Li, W., He, H., Lu, J., Lv, B., Li, M., & Jin, Z. (2013). Evaluation of multiple voxel-based morphometry approaches and applications in the analysis of white matter changes in temporal lobe epilepsy. In H. Liao, C. A. Linte, K. Masamune, T. M. Peters, & G. Zheng (Eds.), *Augmented Reality Environments for Medical Imaging and Computer-Assisted Interventions* (Vol. 8090, pp. 268–276). Berlin, Heidelberg: Springer . http://doi.org/10.1007/978-3-642-40843-4

Liu, D., Johnson, H. J., Long, J. D., Magnotta, V. A., & Paulsen, J. S. (2014). The power-proportion method for intracranial volume correction in volumetric imaging analysis. *Frontiers in Neuroscience*, *8*, 356. http://doi.org/10.3389/fnins.2014.00356

Maclaren, J., Herbst, M., Speck, O., & Zaitsev, M. (2013). Prospective motion correction in brain imaging: A review. *Magnetic Resonance in Medicine*, *69*(3), 621–636. http://doi.org/10.1002/mrm.24314

Malone, I. B., Leung, K. K., Clegg, S., Barnes, J., Whitwell, J. L., Ashburner, J., … Ridgway, G. R. (2015). Accurate automatic estimation of total intracranial volume: A nuisance variable with less nuisance. *NeuroImage*, *104*, 366–372. http://doi.org/10.1016/j.neuroimage.2014.09.034

Mar, R. (2011). The neural bases of social cognition and story comprehension. *Annual Review of Psychology*, *62*, 103–134. http://doi.org/10.1146/annurev-psych-120709-145406

Marsh, A., Stoycos, S., Brethel-Haurwitz, K. M., Robinson, P., VanMeter, J., & Cardinale, E. M. (2014). Neural and cognitive characteristics of extraordinary altruists. *Proceedings of the National Academy of Sciences*, *111*(42), 15036–41. http://doi.org/10.1073/pnas.1408440111

Mechelli, A., Price, C. J., Friston, K., & Ashburner, J. (2005). Voxel-based morphometry of the human brain: Methods and applications. *Current Medical Imaging Reviews*, *1*, 105–113. http://doi.org/10.2174/1573405054038726

Michael, A. M., Evans, E., & Moore, G. J. (2016). Influence of Group on Individual Subject Maps in SPM Voxel Based Morphometry. *Frontiers in Neuroscience*, *10*, 522. http://doi.org/10.3389/fnins.2016.00522

Moessnang, C., Schaefer, A., Bilek, E., Roux, P., Otto, K., Baumeister, S., … Tost, H. (2016). Specificity, reliability and sensitivity of social brain responses during spontaneous mentalizing. *Social Cognitive and Affective Neuroscience*, *11*(11), 1687–1697. http://doi.org/10.1093/scan/nsw098

Molenberghs, P., Johnson, H., Henry, J. D., & Mattingley, J. B. (2016). Understanding the minds of others: A neuroimaging meta-analysis. *Neuroscience and Biobehavioral Reviews*, *65*, 276–291. http://doi.org/10.1016/j.neubiorev.2016.03.020

Morishima, Y., Schunk, D., Bruhin, A., Ruff, C. C., & Fehr, E. (2012). Linking brain structure and activation in temporoparietal junction to explain the neurobiology of human altruism. *Neuron*, *75*(1), 73–79. http://doi.org/10.1016/j.neuron.2012.05.021

Nakamura, K., Brown, R. A., Narayanan, S., Collins, D. L., & Arnold, D. L. (2015). Diurnal fluctuations in brain volume: Statistical analyses of MRI from large populations. *NeuroImage*, *118*, 126–32. http://doi.org/10.1016/j.neuroimage.2015.05.077

O'Brien, L. M., Ziegler, D. A., Deutsch, C. K., Frazier, J. A., Herbert, M. R., & Locascio, J. J. (2011). Statistical adjustments for brain size in volumetric neuroimaging studies: Some practical implications in methods. *Psychiatry Research - Neuroimaging*, *193*(2), 113–122. http://doi.org/10.1016/j.pscychresns.2011.01.007

Otti, A., Wohlschlaeger, A. M., & Noll-Hussong, M. (2015). Is the Medial Prefrontal Cortex Necessary for Theory of Mind? *PLoS ONE*, *10*(8), e0135912. http://doi.org/10.1371/journal.pone.0135912

Prehn, K., Korczykowski, M., Rao, H., Fang, Z., Detre, J. a., & Robertson, D. C. (2015). Neural Correlates of Post-Conventional Moral Reasoning: A Voxel-Based Morphometry Study. *PLoS ONE*, *10*(6), e0122914. http://doi.org/10.1371/journal.pone.0122914

Rice, K., & Redcay, E. (2015). Spontaneous mentalizing captures variability in the cortical thickness of social brain regions. *Social Cognitive and Affective Neuroscience*, *10*(3), 327–34. http://doi.org/10.1093/scan/nsu081

Rice, K., Viscomi, B., & Riggins, T. (2014). Amygdala volume linked to individual differences in mental state inference in early childhood and adulthood. *Developmental Cognitive Neuroscience*, *8*, 153–163. http://doi.org/10.1016/j.dcn.2013.09.003

Ridgway, G. R., Barnes, J., Pepple, T., & Fox, N. (2011). Estimation of total intracranial volume; a comparison of methods. *Alzheimer's & Dementia*, *7*(4), S62–S63. http://doi.org/10.1016/j.jalz.2011.05.099

Ridgway, G. R., Henley, S. M. D., Rohrer, J. D., Scahill, R. I., Warren, J. D., & Fox, N. C. (2008). Ten simple rules for reporting voxel-based morphometry studies. *NeuroImage*, *40*(4), 1429–1435. http://doi.org/10.1016/j.neuroimage.2008.01.003

Ridgway, G. R., Litvak, V., Flandin, G., Friston, K., & Penny, W. D. (2012). The problem of low variance voxels in statistical parametric mapping; a new hat avoids a "haircut." *NeuroImage*, *59*(3), 2131–2141. http://doi.org/10.1016/j.neuroimage.2011.10.027

Ridgway, G. R., Omar, R., Ourselin, S., Hill, D. L. G., Warren, J. D., & Fox, N. C. (2009). Issues with threshold masking in voxel-based morphometry of atrophied brains. *NeuroImage*, *44*(1), 99–111. http://doi.org/10.1016/j.neuroimage.2008.08.045

Sargolzaei, S., Sargolzaei, A., Cabrerizo, M., Chen, G., Goryawala, M., Pinzon-Ardila, A., … Adjouadi, M. (2015). Estimating Intracranial Volume in Brain Research: An Evaluation of Methods. *Neuroinformatics*, *13*(4), 427–41. http://doi.org/10.1007/s12021-015-9266-5

Schurz, M., Radua, J., Aichhorn, M., Richlan, F., & Perner, J. (2014). Fractionating theory of mind: a meta-analysis of functional brain imaging studies. *Neuroscience and Biobehavioral Reviews*, *42*, 9–34. http://doi.org/10.1016/j.neubiorev.2014.01.009

Stadler, A., Schima, W., Ba-Ssalamah, A., Kettenbach, J., & Eisenhuber, E. (2007). Artifacts in body MR imaging: their appearance and how to eliminate them. *European Radiology*, *17*(5), 1242–55. http://doi.org/10.1007/s00330-006-0470-4

Stucht, D., Danishad, K. A., Schulze, P., Godenschweger, F., Zaitsev, M., & Speck, O. (2015). Highest Resolution In Vivo Human Brain MRI Using Prospective Motion Correction. *PLoS ONE*, *10*(7), e0133921. http://doi.org/10.1371/journal.pone.0133921

Thijssen, S., Wildeboer, A., Muetzel, R. L., Bakermans-Kranenburg, M. J., El Marroun, H., Hofman, A., … White, T. (2015). Cortical thickness and prosocial behavior in school-age children: A population-based MRI study. *Social Neuroscience*, *10*(6), 571–582. http://doi.org/10.1080/17470919.2015.1014063

Trefler, A., Sadeghi, N., Thomas, A. G., Pierpaoli, C., Baker, C. I., & Thomas, C. (2016). Impact of time-of-day on brain morphometric measures derived from T1-weighted magnetic resonance imaging. *NeuroImage*, *133*, 41–52. http://doi.org/10.1016/j.neuroimage.2016.02.034

Valk, S., Bernhardt, B., Böckler, A., Trautwein, F.-M., Kanske, P., & Singer, T. (2017). Socio-Cognitive Phenotypes Differentially Modulate Large-Scale Structural Covariance Networks. *Cerebral Cortex*, *27*(2), 1358–1368. http://doi.org/10.1093/cercor/bhv319

Vergara, V. M., Mayer, A., Damaraju, E., Hutchison, K., & Calhoun, V. (2016). The effect of preprocessing pipelines in subject classification and detection of abnormal resting state functional network connectivity using group ICA. *NeuroImage*, *145*, 365–376. http://doi.org/10.1016/j.neuroimage.2016.03.038

Vul, E., & Pashler, H. (2017). Suspiciously high correlations in brain imaging research. In S. O. Lilienfeld & I. D. Waldman (Eds.), *Psychological science under scrutiny: Recent challenges and proposed solutions* (pp. 196–220). New York: Wiley.

Winkler, A. M., Kochunov, P., Blangero, J., Almasy, L., Zilles, K., Fox, P. T., … Glahn, D. C. (2010). Cortical thickness or grey matter volume? The importance of selecting the phenotype for imaging genetics studies. *NeuroImage*, *53*(3), 1135–1146. http://doi.org/10.1016/j.neuroimage.2009.12.028

Yamagishi, T., Takagishi, H., Fermin, A. de S. R., Kanai, R., Li, Y., & Matsumoto, Y. (2016). Cortical thickness of the dorsolateral prefrontal cortex predicts strategic choices in economic games. *Proceedings of the National Academy of Sciences*, *113*(20), 5582–5587. http://doi.org/10.1073/pnas.1523940113

Young, L., Camprodon, J. A., Hauser, M., Pascual-Leone, A., & Saxe, R. (2010). Disruption of the right temporoparietal junction with transcranial magnetic stimulation reduces the role of beliefs in moral judgments. *Proceedings of the National Academy of Sciences*, *107*(15), 6753–6758. http://doi.org/10.1073/pnas.0914826107

Young, L., & Saxe, R. (2008). The neural basis of belief encoding and integration in moral judgment. *NeuroImage*, *40*(4), 1912–20. http://doi.org/10.1016/j.neuroimage.2008.01.057

Young, L., & Saxe, R. (2009). Innocent intentions: A correlation between forgiveness for accidental harm and neural activity. *Neuropsychologia*, *47*(10), 2065–2072. http://doi.org/10.1016/j.neuropsychologia.2009.03.020
